# Evaluation of pulmonary function using single-breath-hold dual-energy computed tomography with xenon: Results of a preliminary study

**DOI:** 10.1097/MD.0000000000005937

**Published:** 2017-01-20

**Authors:** Hiroyuki Kyoyama, Yusuke Hirata, Satoshi Kikuchi, Kosuke Sakai, Yuriko Saito, Shintaro Mikami, Gaku Moriyama, Hisami Yanagita, Wataru Watanabe, Katharina Otani, Norinari Honda, Kazutsugu Uematsu

**Affiliations:** a Department of Pulmonary Medicine; b Department of Radiology, Saitama Medical Center, Saitama Medical University, Saitama; c Research and Collaborations Department, Siemens Healthcare KK, Tokyo, Japan.

**Keywords:** chronic obstructive pulmonary disease, contrast media, dual-energy computed tomography, pulmonary function test, single-breath, xenon

## Abstract

Xenon-enhanced dual-energy computed tomography (xenon-enhanced CT) can provide lung ventilation maps that may be useful for assessing structural and functional abnormalities of the lung. Xenon-enhanced CT has been performed using a multiple-breath-hold technique during xenon washout. We recently developed xenon-enhanced CT using a single-breath-hold technique to assess ventilation. We sought to evaluate whether xenon-enhanced CT using a single-breath-hold technique correlates with pulmonary function testing (PFT) results.

Twenty-six patients, including 11 chronic obstructive pulmonary disease (COPD) patients, underwent xenon-enhanced CT and PFT. Three of the COPD patients underwent xenon-enhanced CT before and after bronchodilator treatment. Images from xenon-CT were obtained by dual-source CT during a breath-hold after a single vital-capacity inspiration of a xenon–oxygen gas mixture. Image postprocessing by 3-material decomposition generated conventional CT and xenon-enhanced images.

Low-attenuation areas on xenon images matched low-attenuation areas on conventional CT in 21 cases but matched normal-attenuation areas in 5 cases. Volumes of Hounsfield unit (HU) histograms of xenon images correlated moderately and highly with vital capacity (VC) and total lung capacity (TLC), respectively (*r* = 0.68 and 0.85). Means and modes of histograms weakly correlated with VC (*r* = 0.39 and 0.38), moderately with forced expiratory volume in 1 second (FEV_1_) (*r* = 0.59 and 0.56), weakly with the ratio of FEV_1_ to FVC (*r* = 0.46 and 0.42), and moderately with the ratio of FEV_1_ to its predicted value (*r* = 0.64 and 0.60). Mode and volume of histograms increased in 2 COPD patients after the improvement of FEV_1_ with bronchodilators. Inhalation of xenon gas caused no adverse effects.

Xenon-enhanced CT using a single-breath-hold technique depicted functional abnormalities not detectable on thin-slice CT. Mode, mean, and volume of HU histograms of xenon images reflected pulmonary function. Xenon images obtained with xenon-enhanced CT using a single-breath-hold technique can qualitatively depict pulmonary ventilation. A larger study comprising only COPD patients should be conducted, as xenon-enhanced CT is expected to be a promising technique for the management of COPD.

## 1 Introduction

Spirometry is currently the standard method for evaluating pulmonary function, but testing is highly dependent on the subject's cooperation, as it requires the use of a mouthpiece, response to verbal commands, and an effort to breathe deeply. Further, while spirometry provides a global functional evaluation of the lungs, the location of pulmonary abnormalities cannot usually be deduced. To improve these shortcomings, imaging modalities such as high-resolution computed tomography (CT) and magnetic resonance imaging have been considered.^[1,2]^ The extent of emphysema and assessment of airways on CT images have been shown to correlate with pulmonary function,^[1]^ but distinguishing areas of impaired function from normal areas on CT images remains difficult.

Nonradioactive xenon gas has been used to assess cerebral blood flow on conventional CT^[3]^ and has been proposed as a contrast medium for the diagnosis of lung diseases due to its high density and higher attenuation of X-rays than air. Recently, dual-energy CT has allowed simultaneous acquisition of data at 2 different tube voltages, thereby permitting differentiation of materials on postprocessed images (eg, differentiation of xenon from air and soft tissue). Postprocessing by 3-material decomposition of dual-energy-CT data taken after inhalation of xenon (xenon-enhanced dual-energy computed tomography [xenon-enhanced CT]) can therefore provide nonenhanced and xenon-enhanced image data. Xenon-enhanced CT has thus emerged as a promising technique for the detection of abnormal lung ventilation, allowing the assessment of structural as well as functional abnormalities of the lung.^[4]^

Xenon-enhanced CT has been reported to be useful for the detection of obstructive lung diseases including chronic obstructive pulmonary disease (COPD), bronchial asthma, and bronchiolitis obliterans.^[5–11]^ In these studies, dynamic data were acquired by xenon-enhanced CT using a multiple-breath-hold technique during xenon washout. We recently used xenon-enhanced CT with a single-breath-hold technique to assess ventilation.^[12]^ Using this method, we were able to predict residual pulmonary function after lung resection for cancer.^[13]^

Here, we evaluated whether xenon CT using single-breath-hold technique correlates with pulmonary function test (PFT) results. We also assessed the usefulness of xenon-enhanced CT for the detection of response to therapeutic agents for COPD.

## 2 Materials and methods

### 1.1 Patients

Patients referred to our department for suspected obstructive pulmonary diseases between 2011 and 2014 and who gave consent were enrolled in this study. A 33-year-old and a 66-year-old male volunteer with no history of smoking and respiratory diseases participated as healthy controls. The trial was approved by the ethics board of our hospital and registered to the University Hospital Medical Information Network in Japan (ID: UMIN 000006850). Informed consent was obtained from all patients. Patients who were already under treatment with bronchodilators or were aged under 20 years were excluded.

Patients enrolled in the study underwent both xenon-enhanced CT and PFT. COPD was diagnosed in accordance with the criteria of the Global Initiative for Chronic Obstructive Lung Disease.^[14]^ COPD patients underwent xenon-enhanced CT before and after treatment with bronchodilators.

### 1.2 Pulmonary function tests

PFT consisted of measurements of vital capacity (VC), forced vital capacity (FVC), total lung capacity (TLC), forced expiratory volume in 1 second (FEV_1_), maximal expiratory flow at 50% and 25% of the FVC  
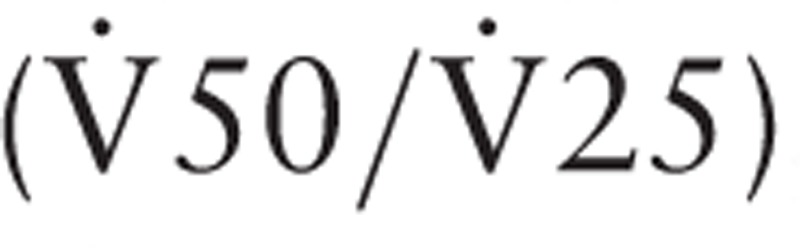
, and the diffusing capacity divided by the alveolar volume (DLCO/VA). All tests were done in the seated position using a dry rolling-seal spirometer (CHESTAC-9800; Chest, Tokyo, Japan). Predicted FEV_1_ was calculated using the prediction equation of the Japanese Respiratory Society in 2001.^[15]^ The ratio of FEV_1_ to FVC (FEV_1_/FVC) and FEV_1_ as a percentage of predicted (FEV_1_% pred) were calculated.

### 1.3 Xenon images and histograms

Xenon-enhanced CT was performed within 1 week of PFT. We followed our standard scanning method.^[12,13]^ Briefly, patients in the supine position were scanned in dual-energy mode during breath-hold after a single vital-capacity (from maximum expiration to maximum inspiration) inhalation of a mixture of xenon and oxygen in a ratio of 35:65 using a dual-source CT scanner (SOMATOM Definition Flash; Siemens Healthcare, Forchheim, Germany). Scanning conditions were as follows: tube voltages, 140 kV filtered with 0.4-mm-thick tin and 80 kV; beam collimation, 0.6 mm × 128 rows; pitch, 0.55; reconstruction section thickness, 1.5 mm; interval, 1 mm; and reconstruction kernel, D30f medium smooth. The 35% xenon gas was prepared and stored in a xenon gas supplier (Az-726V Xetron VI; Anzai Medical Corporation, Tokyo, Japan). The effective radiation dose was estimated from the dose-length product automatically enumerated by the CT scanner and a conversion factor of 0.018.^[16]^ Sets of xenon-enhanced images and standard lung CT images were generated by 3-material decomposition on a dedicated workstation (Syngo MMWP; Siemens Healthcare, Forchheim, Germany). Parameters of the 3-material decomposition were set to the default of the manufacturer. Segmentation of the lung from xenon images was performed automatically during decomposition, after which the set was archived to another workstation (AZE Virtual Place Plus; AZE Corporation, Tokyo, Japan) and analyzed as below.

On xenon images covering the whole lung for each patient, the total numbers of pixels of the lung were counted. Frequency histograms were plotted with the number of pixels on the ordinate and the corresponding pixel value on the abscissa starting from the minimum to the maximum pixel value. The small peak on the left side of the histogram was omitted from analysis after confirming that these pixels corresponded to signal from the pulmonary veins. Since the area under the curve is equal to the number of voxels within the lung contour of the set, the sum was converted to lung volume by multiplying the square of pixel pitch and slice interval of the set. The mean in Hounsfield units (HUs) and the mode as most frequent value were calculated for each patient's histogram.

### 1.4 Statistics

Linear correlation analysis was applied to estimate correlations between the volume and VC or TLC, between the mean and FEV_1_/FVC or FEV_1_% pred, and between the mode and FEV_1_/FVC or FEV_1_% pred. These correlations were analyzed using patient's data, excluding the data of the healthy volunteers.

Statistical analysis was performed with a commercial statistical software package (SPSS Statistics 21; IBM, Armonk, NY). Correlations were evaluated based on Pearson correlation coefficients. *P* values < 0.05 were considered statistically significant.

## 3 Results

All 26 enrolled patients were eligible for this study. These patients and 2 healthy volunteers underwent both xenon-enhanced CT and PFT. Eleven were diagnosed with COPD, 3 showed combined pulmonary fibrosis and emphysema, and 12 had other lung diseases (Table 1). Inhalation of xenon gas was not associated with any adverse effects. Three COPD patients underwent xenon-enhanced CT again within 2 to 6 months after beginning treatment with bronchodilators.

**Table 1:**
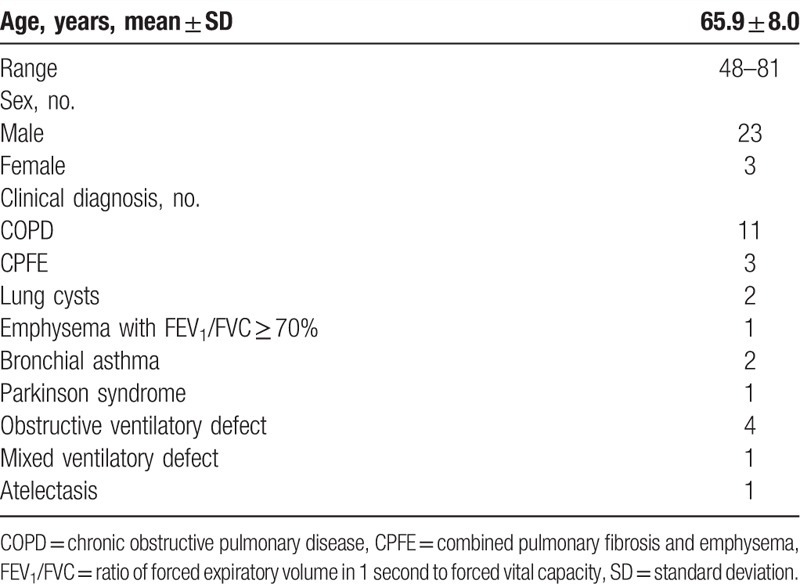
Clinical characteristics of patients (no. = 26).

**Figure 1:**
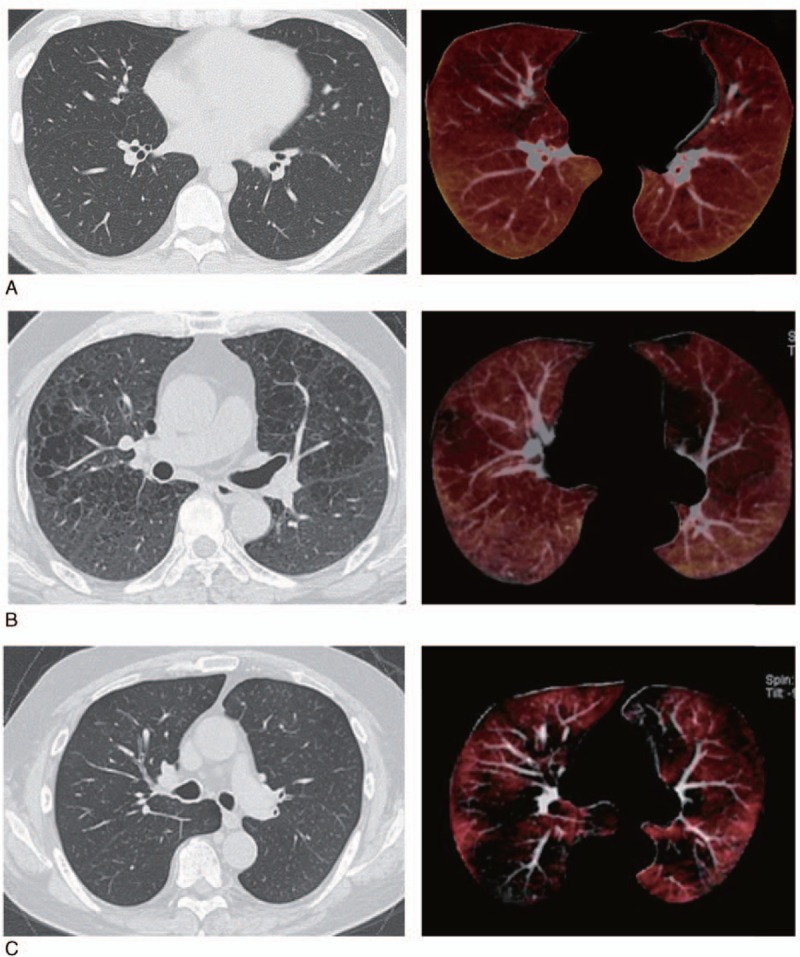
Comparison of conventional or thin-slice CT (left) and xenon images (right). (A) A 33-year-old healthy male, nonsmoker. Conventional CT and pulmonary function test results were within normal limits. (B) A 69-year-old male with COPD. The FEV_1_/FVC ratio was 62%. Pulmonary emphysema is depicted as low-attenuation areas on thin-slice CT (left) corresponding to areas of low attenuation on xenon CT (right). (C) A 66-year-old male with COPD. The FEV_1_/FVC was 33%. Xenon images (right) show low-attenuation areas which are not seen on thin-slice CT (left). COPD = chronic obstructive pulmonary disease, CT = computed tomography, FEV_1_/FVC = ratio of forced expiratory volume in 1 second to forced vital capacity.

The xenon images of the 2 healthy volunteers with normal pulmonary function showed homogenous lung density without focal areas of increased or decreased density than surrounding lung parenchyma. These images showed gradual increase in density from anterior to posterior in accordance with gravity (Fig. 1). Low-attenuation areas on conventional CT corresponded to areas of low HU on xenon images. However, additional low-density areas on xenon images corresponded to normal areas on conventional or thin-slice CT in 5 patients but matched those on conventional CT in the remaining 21 patients (Fig. 1). These 5 mismatched patients comprised 3 cases of COPD, 1 case of bronchial asthma, and 1 case of bronchiolitis. The COPD and asthma patients were smokers (Table 2).

**Table 2:**
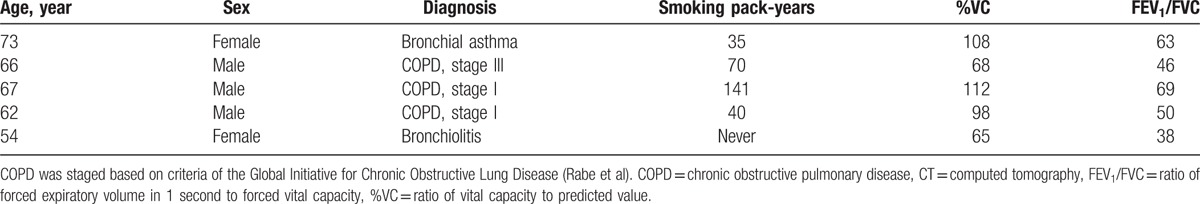
Characteristics of patients with ventilation abnormalities on xenon images which were not detectable on high-resolution CT.

**Figure 2:**
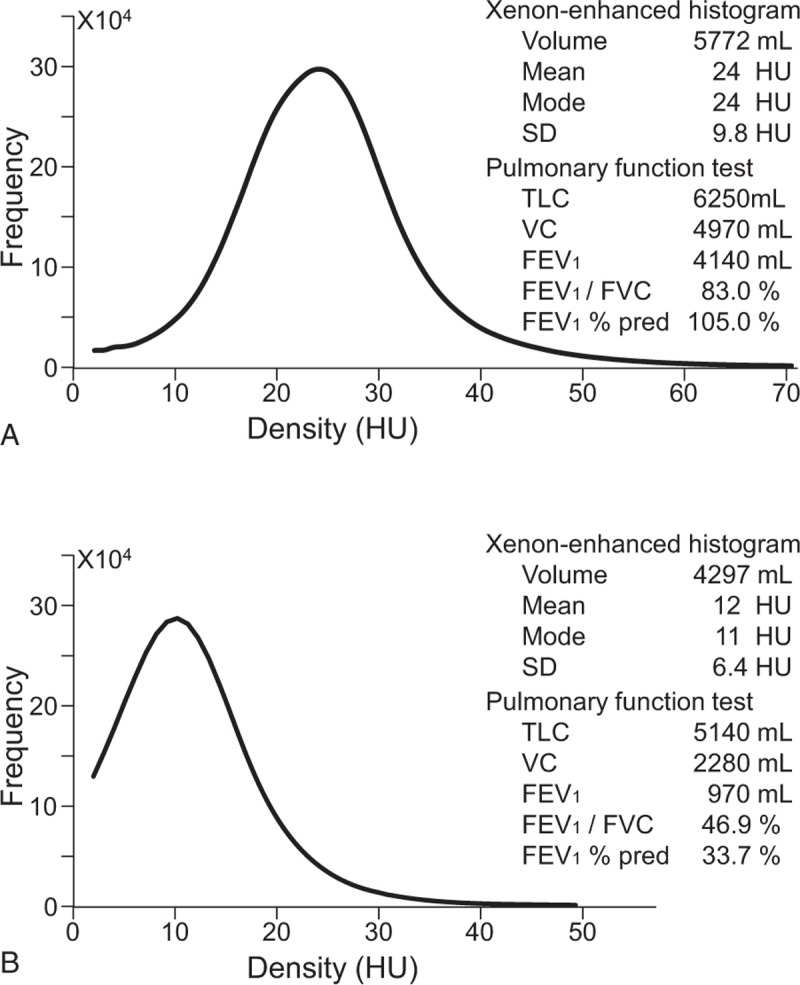
Xenon histogram. (A) Density histogram of the xenon images of a 33-year-old healthy male with normal pulmonary function. (B) Density histogram of the xenon images of a 66-year-old patient with COPD stage III. The mode of the histogram was shifted to the left compared to that of a patient with better FEV_1_/FVC and FEV_1_% pred. COPD = chronic obstructive pulmonary disease, FEV_1_/FVC = ratio of forced expiratory volume in 1 second to forced vital capacity, FEV_1_% pred = FEV_1_ as a percentage of predicted.

Twenty-nine histograms from CT images of 26 patients, 3 of whom underwent xenon-enhanced CT and PFT before and after the treatment of COPD, and 2 histograms from 2 healthy volunteers were generated. All histograms had a small peak on the left side, consisting of signal from the pulmonary veins, and a threshold of 2 HU was found to be appropriate to remove this peak. The mode of the histograms of severe COPD patients was shifted to the left compared to that of healthy controls. Figure 2 shows example histograms from a 33-year-old healthy volunteer with normal pulmonary function and a 66-year-old patient with severe COPD.

PFT of the patients gave the following results: FEV_1_/FVC ranged from 38.9% to 88.1%, with 18 patients having values below 70%. The means and ranges of TLC, VC, FEV_1_, DLCO/VA, and  
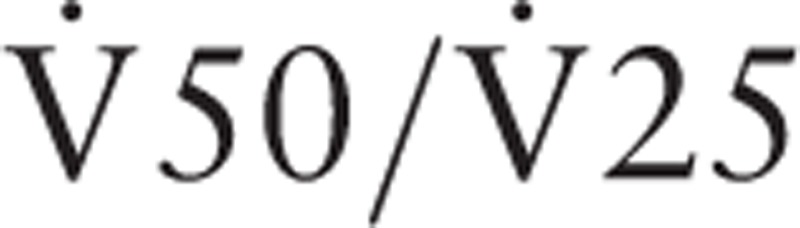
 were 5577 mL (range, 2850–8410 mL), 3622 mL (range, 1730–5440 mL), 2305 mL (range, 650–3360 mL), 3.9 mL/min/mm Hg/L (range, 1.9–5.99 mL/min/mm Hg/L), and 4.44 (1.71–8.25), respectively. The correlation between basic statistics of xenon-enhanced CT and pulmonary function test were evaluated using 29 histograms from 26 patients.

**Figure 3:**
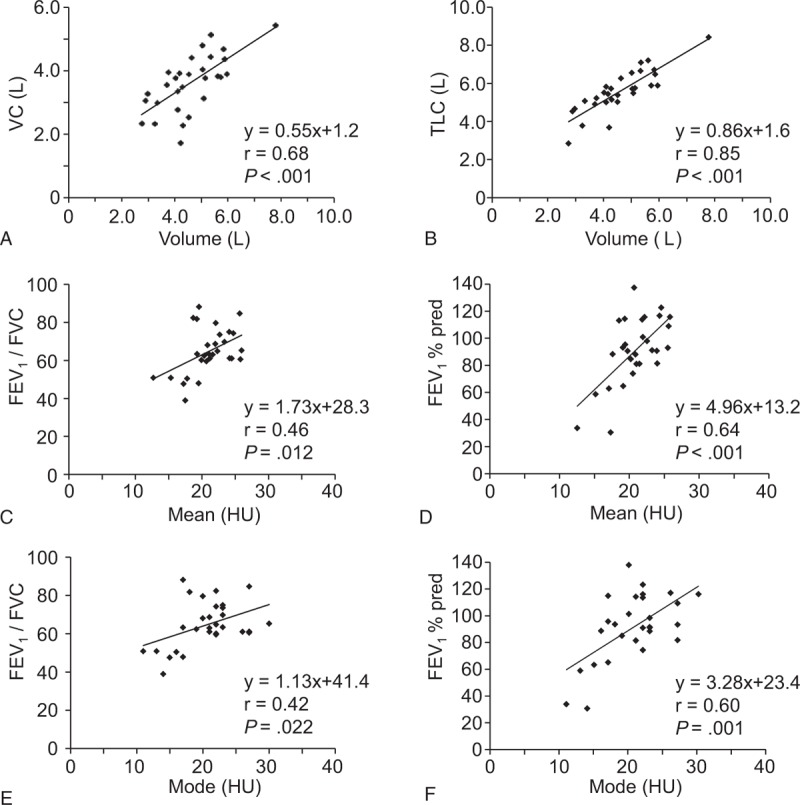
Relationship between basic statistics of xenon-enhanced CT and pulmonary function tests of the patients. Volume, calculated as area under the curve, of the density histogram of xenon images was correlated to VC (A) and TLC (B). The mean of the histogram was correlated to FEV_1_/FVC (C) and FEV_1_% Pred (D), and the mode was also correlated to FEV_1_/FVC (E) and FEV_1_% pred (F). CT = computed tomography, FEV_1_/FVC = ratio of forced expiratory volume in 1 second to forced vital capacity, FEV_1_% pred = FEV_1_ as a percentage of predicted, TLC = total lung capacity, VC = vital capacity, xenon-enhanced CT = xenon-enhanced dual-energy computed tomography.

Volumes correlated moderately with VC and correlated highly with TLC (*r* = 0.68 and 0.85, respectively) (Fig. 3A, B). Means weakly correlated with VC (*r* = 0.39), and moderately with FEV_1_, FEV_1_/FVC, and FEV_1_% pred (*r* = 0.59, 0.46, and 0.64, respectively) (Fig. 3C, D). Modes of densities on histograms of xenon images weakly correlated with VC (*r* = 0.38), moderately with FEV_1_, FEV_1_/FVC, and FEV_1_% pred (*r* = 0.56, 0.42, and 0.60, respectively) (Fig. 3E, F). TLC was strongly correlated with the volume of a histogram but did not correlate at all with mean or mode (*r* = 0.03 and 0.09, respectively). None of the parameters correlated with DLCO/VA or  
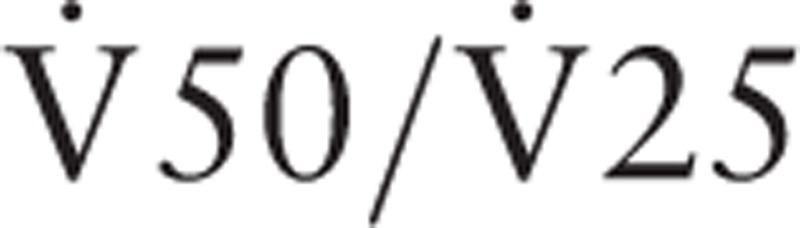
.

**Figure 4:**
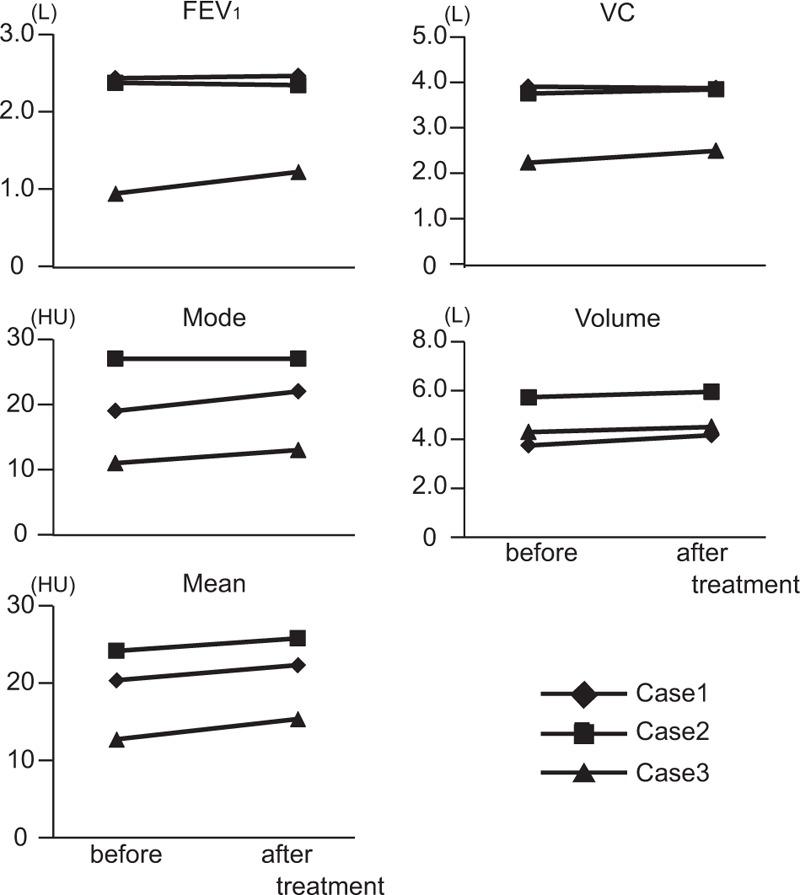
Changes in basic statistics of xenon-enhanced CT and pulmonary function tests by treatment for COPD. Xenon CT and pulmonary function tests were performed before and after treatment with bronchodilators in three COPD patients. Cases 1 (diamond) and 3 (triangle) show increase in FEV_1_, and the mode and volume of the histogram were shifted to the right after treatment. COPD = chronic obstructive pulmonary disease, CT = computed tomography, FEV_1_ = forced expiratory volume in 1 second, xenon-enhanced CT = xenon-enhanced dual-energy computed tomography.

On histograms of the 3 COPD patients who underwent a 2nd round of xenon-enhanced CT after starting treatment with bronchodilators, the mode, mean, and volume all increased in 2 patients in accordance with an increase in FEV_1_ (Fig. 4, cases 1 and 3), while the mean and volume of the remaining patient, who showed no significant changes in FEV_1_ (from 2400 to 2370 mL), increased only slightly and the mode was unchanged.

## 4 Discussion

In this pilot study, we evaluated the usefulness of xenon-enhanced CT images acquired with a single-breath-hold technique and compared our findings with PFT. We found a correlation between the statistical indices (volume, mean, and mode) of the density histograms of xenon images and PFT results. Volumes moderately and strongly correlated with VC and TLC of PFTs, respectively, while means and modes weakly correlated with VC and FEV_1_/FVC, and moderately with FEV_1_ and FEV_1_% pred, respectively. These results were not affected when 2 healthy volunteers’ data were included. Of note, FEV_1_% pred, which is used to determine COPD severity,^[14]^ correlated more closely to the means and modes of the histograms, suggesting that xenon images may be useful for grading the severity of COPD.

Xenon-enhanced CT simultaneously provides a conventional or thin-slice unenhanced image and a xenon-enhanced image. Areas of reduced ventilation are expected to be depicted as areas of lower density relative to areas of normal ventilation on xenon images. We found that low-attenuation areas on conventional or thin-slice CT corresponded to areas of low density on xenon-enhanced images; however, 5 patients had low-density areas in xenon-enhanced images which looked normal in thin-slice unenhanced images, suggesting areas of reduced ventilation may not always correspond to low-density areas on thin-slice unenhanced CT. The density distributions on xenon-enhanced images may thus reveal additional ventilation abnormalities in apparently normal areas on unenhanced CT.

COPD is characterized by incompletely reversible airflow limitation resulting from a mixture of obstructive small airway disease and emphysematous lung tissue destruction.^[14]^ Emphysema severity assessed by CT has been reported to predict worsening lung function^[17,18]^ and increased mortality.^[19]^ Xenon-enhanced imaging is expected to be more sensitive than unenhanced thin-slice CT for identifying emphysematous areas and related areas of obstructed airflow. This method may therefore be able to detect obstructive ventilatory impairment and disproportionate ventilation. In COPD, inspiratory and expiratory CT have been used to assess air trapping due to small airway obstruction.^[2,20–22]^ Future studies should compare findings on xenon-enhanced CT with those using the inspiratory/expiratory unenhanced CT method.

When 3 patients with COPD underwent repeat xenon-enhanced CT after starting bronchodilator therapy, the modes and means increased in the 2 COPD patients who showed increases in FEV_1_ after treatment. Bronchodilators relieve air-trapping or ameliorate disproportionate ventilation, thereby improving patients’ dyspnea and exercise capacity. However, FEV_1_ often fails to reflect these improvements despite the improvement in a patient's condition. Modes or means of density histograms of xenon images might correlate with severity of air-trapping or disproportionate ventilation in our study and could thus function as an indicator for the evaluation of COPD treatment effect.

We expect the clinical importance of xenon-enhanced CT compared to conventional PFT as follows. PFT allows global assessment of lung volumes and ventilation, while xenon-enhanced CT depicts regional alteration of ventilation as images, and simultaneously allows global assessment of ventilation as quantitative values. Findings from our study also suggested that the volume of density histograms of xenon images may be an index of restrictive ventilatory impairment and modes and means may be indices of obstructive ventilatory disturbances, such as uneven flow in airways.

Recent studies have described the entity of combined pulmonary fibrosis and emphysema, which is a disease not adequately evaluated by PFT.^[23]^ Patients with this disease show emphysema of the upper lung zones and diffuse fibrosis in the lower lung zones on conventional chest CT, as well as severe impairment of gas exchange and poor survival. However, despite the severity of these patients’ conditions, TLC, FVC, FEV_1_, and FEV_1_% pred show only moderate impairment. We expect that xenon imaging could allow a more precise diagnosis of patients such as these. Future studies designed to prove these assumption are necessary.

The limitations of our study were the small number of patients and the variety of lung diseases afflicting our population. A larger study comprising only COPD patients should be conducted, as xenon-enhanced CT may be a promising technique for the management of COPD in particular.

Dual-energy xenon-enhanced CT using a single-breath-hold technique was found feasible and safe for patients with various lung diseases. Our study indicated that xenon images acquired with dual-energy CT in the single-breath-hold technique and processed with 3-material decomposition can qualitatively depict pulmonary ventilation. Further, xenon-enhanced CT images revealed ventilation abnormalities not detectable on conventional or thin-slice CT. The volume, mean, and mode of histograms generated from densities on xenon images correlated with PFT results (VC, FEV_1_, FEV_1_/FVC, and FEV_1_% pred) and may be useful parameters for diagnosing lung disease and detecting response to therapeutic agents in COPD patients.

## Acknowledgments

The authors thank Siemens Japan KK for the support.
